# Construction and validation of a machine learning model for predicting anastomotic leak following radical esophagectomy for esophageal cancer

**DOI:** 10.3389/fonc.2026.1877476

**Published:** 2026-07-24

**Authors:** Yueying Yang, Kayishaer Ainiwaer, Yunfei Gao, Ao Li, Abulajiang Kamili, Dongbo Luo

**Affiliations:** 1 Department of Thoracic Surgery II, Xinjiang Medical University Affiliated Tumor Hospital, Urumqi, Xinjiang, China; 2 The Third Clinical Medical College of Xinjiang Medical University, Urumqi, Xinjiang, China

**Keywords:** aL, EC, esophagectomy, ML, preoperative prediction, RF

## Abstract

**Background:**

Early identification of patients at high risk of anastomotic leak (AL) following esophagectomy is essential for improving surgical outcomes. However, reliable preoperative risk stratification remains challenging. This study aimed to predict AL risk in the esophageal cancer (EC) population by developing and validating a machine learning (ML)-based model using exclusively preoperative and baseline clinical data.

**Methods:**

A retrospective cohort of EC patients who underwent radical esophagectomy at the Affiliated Tumor Hospital of Xinjiang Medical University from January 2020 to May 2025 was analyzed. Feature selection was performed using the least absolute shrinkage and selection operator (LASSO) regression. Five ML algorithms, Random Forest (RF), Support Vector Machine (SVM), Logistic Regression (LR), Extreme Gradient Boosting (XGBoost), and Light Gradient Boosting Machine (LightGBM), were constructed. Model performance was comprehensively evaluated using the area under the receiver operating characteristic (ROC) curve (AUC), calibration curves, and decision curve analysis (DCA). Model interpretability was enhanced via Shapley Additive Explanations (SHAP), enabling quantification of feature importance and visualization of individual prediction contributions.

**Results:**

A total of 368 patients were included and randomized to training (n=258) and validation (n=110) cohorts. Among the models, RF displayed the best discriminative performance in the validation cohort (AUC: 0.803, 95% confidence interval [CI]: 0.717-0.892), followed by XGBoost (AUC: 0.723) and LightGBM (AUC: 0.713). The RF model yielded a sensitivity (SEN) of 0.879 and a specificity (SPE) of 0.571. SHAP analysis identified monocytes, carcinoembryonic antigen (CEA), neutrophil-to-lymphocyte ratio (NLR), urine creatinine (UCr), and T stage as the five most influential predictors of AL. Calibration curves for the ensemble models demonstrated good agreement between predicted probabilities and observed outcomes.

**Conclusions:**

The RF model, incorporating five routinely available preoperative variables, exhibited robust discriminative performance with high SEN for predicting in-hospital AL following esophagectomy. The proposed threshold-based risk stratification approach may facilitate individualized perioperative monitoring and management.

## 1 Background

Esophageal cancer (EC) ranks as the seventh most common malignancy worldwide, with approximately 600,000 new cases and 540,000 deaths annually (1). Its global incidence has increased sharply (2, 3). Considerable geographical variation exists, with East Asia bearing a disproportionately high burden (4, 5). According to GLOBOCAN 2022, both the incidence and mortality of EC are projected to continue increasing through 2050. Although patient survival has improved over the past two decades owing to multimodal treatment strategies incorporating surgery, chemotherapy, radiotherapy, and immunotherapy, the prognosis of EC remains poor. The five-year overall survival rate across all stages is approximately 20% (6, 7). Therefore, EC has emerged as a substantial oncological burden globally.

Surgery remains the cornerstone of curative treatment for resectable EC and plays a critical role in prolonging survival and improving quality of life, particularly among patients with locally advanced disease. It provides the greatest opportunity for cure in patients with early-stage disease and remains the primary treatment for relieving tumor-related dysphagia and controlling local tumor progression. Common surgical procedures include transthoracic Ivor-Lewis esophagectomy and the McKeown procedure. However, esophagectomy is among the most technically demanding procedures in thoracic surgery because it involves extensive thoracoabdominal dissection, organ resection, and complex gastrointestinal reconstruction. It is associated with a postoperative complication rate of 35%-50%, including anastomotic leak (AL), recurrent laryngeal nerve injury, atrial fibrillation, chylothorax, and pneumonia (8). These complications increase patient morbidity and healthcare costs and adversely affect both clinical outcomes and long-term prognosis. AL is among the most severe postoperative complications and is defined as a loss of anastomotic integrity resulting in leakage of luminal contents, with a reported incidence ranging from 5% to 30% (9–11). Although minimally invasive surgery has reduced overall complications, the risk of AL remains (12). Kamarajah et al. reported that AL increases 90-day mortality by three- to fourfold and reduces five-year overall survival by 15%-20% (13). Mechanistically, AL may adversely affect prognosis through multiple pathways, including the induction of systemic inflammation and alterations in the tumor microenvironment. According to the International Society for Diseases of the Esophagus, AL is classified as a “critical surgery-related event impacting survival” (10, 13, 14). Current risk assessment for AL largely relies on conventional scoring systems or logistic regression (LR)-based nomograms, which assume linear relationships and may fail to capture complex nonlinear interactions among risk factors. Machine learning (ML) algorithms can model high-dimensional nonlinear relationships and have demonstrated promise in predicting surgical outcomes.

Given the markedly increased risk of adverse outcomes associated with AL and the lack of effective predictive tools in routine clinical practice, early identification of high-risk patients remains challenging. Therefore, this retrospective study explored the feasibility of developing an ML-based prediction model using interpretable clinical features to predict AL and support future clinical decision-making.

## 2 Methods

### 2.1 Study design and population

This retrospective cohort study included patients who underwent radical esophagectomy for EC at the Affiliated Tumor Hospital of Xinjiang Medical University between January 2020 and May 2025 and was conducted in accordance with the Transparent Reporting of a Multivariable Prediction Model for Individual Prognosis or Diagnosis Plus Artificial Intelligence (TRIPOD+AI) reporting guideline.

Inclusion criteria were: (i) histologically confirmed EC; (ii) age 42–84 years; (iii) curative-intent radical esophagectomy; (iv) definitive in-hospital determination of AL status according to a standardized diagnostic protocol (Section 2.3); and (v) complete preoperative clinical and laboratory data. Exclusion criteria were: (i) concurrent malignancy or distant metastasis; (ii) emergency or palliative resection; (iii) severe preoperative organ dysfunction (Child-Pugh class C liver disease, New York Heart Association (NYHA) class III-IV heart failure, or estimated glomerular filtration rate (eGFR) <30 mL/min/1.73 m²); and (iv) incomplete preoperative data or loss to follow-up before discharge. Importantly, patients with clinically meaningful Esophagectomy Complications Consensus Group (ECCG) type II or type III AL, including severe AL requiring interventional, endoscopic, or surgical management, were not excluded. These cases were retained in the AL group to preserve the clinical representativeness and severity spectrum of the outcome.

The Institutional Review Board of the Affiliated Tumor Hospital of Xinjiang Medical University approved the study (Approval No.: K-2025072). Patient data were fully anonymized. All patients provided written informed consent before surgery. Additional consent was not required for this retrospective analysis.

Following multidisciplinary evaluation, all patients underwent radical esophagectomy performed by board-certified thoracic surgeons with more than five years of experience in esophageal surgery. The surgical approach was determined according to tumor location: McKeown three-field esophagectomy for upper thoracic tumors and McKeown, Ivor-Lewis, or Sweet procedures for middle or lower thoracic tumors. Standardized procedures included complete esophageal mobilization, mediastinal lymphadenectomy, gastric conduit construction using a linear stapler, preservation of the right gastroepiploic vascular arcade with an ultrasonic scalpel, and cervical or intrathoracic esophagogastric anastomosis.

### 2.2 Covariates

Based on previous studies and clinical experience, the following variables were collected: (i) demographic characteristics (sex and age); (ii) anthropometric measurements (height and weight); (iii) smoking history, alcohol consumption, hypertension, and diabetes mellitus; (iv) tumor-related variables (distance from the incisors measured by preoperative gastroscopy, distance-to-height ratio, and neoadjuvant therapy status); (v) operative variables collected for descriptive purposes only (operative time and intraoperative blood loss); (vi) clinical tumor-node-metastasis (TNM) stage according to the American Joint Committee on Cancer (AJCC) 8th edition staging system; (vii) tumor characteristics (location, gross morphology, histological subtype, and grade); and (viii) laboratory parameters, including complete blood count (hemoglobin, red blood cell count, and white blood cell count), differential leukocyte count (neutrophils, lymphocytes, and monocytes), renal function indicators (urine creatinine (UCr) and serum creatinine (SCr)), liver function indicators (albumin, alanine aminotransferase (ALT), aspartate aminotransferase (AST), alkaline phosphatase (ALP), and total bilirubin), metabolic indicators (eGFR, total cholesterol, triglycerides, cystatin C, and blood glucose), electrolytes (sodium [Na^+^] and potassium [K^+^]), coagulation parameters, and tumor markers including carcinoembryonic antigen (CEA). Postoperative complications such as pulmonary infection were not considered candidate predictors in the final preoperative prediction model.

### 2.3 Diagnostic criteria for outcome variable

The primary outcome was the incidence of post-esophagectomy AL during the index hospitalization, defined according to ECCG criteria as a full-thickness defect involving the esophagus, anastomosis, staple line, or conduit, irrespective of clinical presentation or method of detection. AL was classified as ECCG type I, II, or III according to the treatment required: type I requiring no specific intervention or dietary modification alone, type II requiring interventional but not surgical treatment, and type III requiring surgical treatment. No clinically meaningful ECCG type II or III leaks were excluded from the analysis. All patients underwent routine postoperative clinical assessment, drain-output monitoring, and thoracoabdominal computed tomography (CT) approximately one week after surgery. Radiographic findings suggestive of AL included free air, mediastinal or cervical fluid collections, contrast extravasation, or pleural effusion suspicious for anastomotic disruption. Cervical AL typically presented with neck swelling, tenderness, and purulent discharge containing food debris or saliva after wound opening. Thoracic AL commonly manifests as pleural effusion, chest pain, fever, systemic inflammatory response, or drainage of gastric contents or pus through the chest tube. Upper gastrointestinal endoscopy and/or water-soluble contrast esophagography were performed when clinically indicated to confirm suspected AL. The diagnostic time window was restricted to the postoperative in-hospital period.

### 2.4 Missing value imputation

Variables with more than 25% missing values were excluded from the candidate predictor pool. For the remaining variables, all of which had less than 15% missingness, missing values were imputed using multiple imputation by chained equations (MICE), with 10 iterations and 5 imputations, implemented using the mice package (version 3.16.0) in R. The imputation model included all candidate predictors and the outcome variable (AL status) to preserve underlying relationships among variables and minimize imputation bias. Imputation was performed on the full dataset before the 70:30 stratified random split, generating complete datasets for subsequent feature selection and model development. Although the overall missing-data rate was low (<5%) and the training and validation cohorts were randomly sampled from the same source population, performing imputation before data partitioning may have introduced potential information leakage. This issue is therefore acknowledged as a limitation in the Discussion section. In future validation studies, imputation models should be developed using only the training dataset and subsequently applied to validation or external datasets to further minimize the risk of information leakage.

### 2.5 Statistical methods

The normality of continuous variables was assessed via the Shapiro-Wilk test. Normally distributed variables were shown in mean ± standard deviation (SD), with between-group comparisons (training vs validation sets) performed using independent-samples t tests. Non-normally distributed ones were shown in medians with interquartile ranges (IQRs) and compared employing the Mann-Whitney U test. Categorical ones were in counts and percentages, and group differences were examined via the chi-square or Fisher’s exact test (when expected cell counts were<5). Two-sided P<0.05 signified statistical significance.

### 2.6 Data partitioning

The overall cohort was randomized into training (70%) and validation (30%) sets utilizing stratified random sampling based on AL status, ensuring preservation of event proportions across both subsets.

### 2.7 Feature selection

To reduce overfitting and identify the most informative predictors from the 56 candidate variables, the least absolute shrinkage and selection operator (LASSO) LR was applied to the training set. This method performs simultaneous variable selection and regularization through an L1 penalty, shrinking coefficients with minimal predictive value toward zero. The regularization parameter (λ) was optimized using 10-fold cross-validation, and the optimal model was selected according to the minimum cross-validated binomial deviance (lambda.min).

### 2.8 Model development

Five ML algorithms were trained using the predictors selected by LASSO. The same set of LASSO-selected features was used across all models to maintain a consistent predictor space and enable a fair comparison of algorithmic performance: (i) LR: A generalized linear model used as the baseline comparator, incorporating L2 regularization (ridge penalty) to improve coefficient stability. (ii) Random Forest (RF): A bagging-based ensemble method that constructs multiple decorrelated decision trees using bootstrap samples and random feature selection at each split, with predictions aggregated through majority voting. (iii) Support Vector Machine (SVM): A kernel-based classifier employing a radial basis function (RBF) kernel to project features into a higher-dimensional space for nonlinear classification. (iv) Support Vector Machine (SVM) (XGBoost): A gradient boosting algorithm that sequentially constructs decision trees while incorporating L1/L2 regularization and column subsampling to reduce overfitting. (v) Light Gradient Boosting Machine (LightGBM): A gradient boosting framework that adopts leaf-wise tree growth and histogram-based feature binning to improve computational efficiency.

Model development and statistical analyses were performed using R 4.5.2. The primary R packages included mice 3.16.0 for multiple imputation, glmnet 4.1–4 for LASSO regression and LR, randomForest 4.7-1.1 for RF modeling, e1071 1.7–13 for SVM, xgboost 1.7.5 for XGBoost, lightgbm 3.3.5 for LightGBM, caret 6.0–93 for model training and workflow coordination, pROC for ROC analysis, and Shapley Additive Explanations (SHAP)-related packages for model interpretation. The analytical code is available from the corresponding author upon reasonable request.

### 2.9 Model evaluation and interpretability

Model discrimination was evaluated using the area under the receiver operating characteristic (ROC) curve (AUC). The 95% CIs were estimated using the DeLong method. Optimal classification thresholds were determined by maximizing Youden’s index (sensitivity (SEN) + specificity (SPE) − 1), from which the corresponding SEN and SPE values were calculated. The best-performing model was selected based on the highest AUC in the validation set, with emphasis on generalizability. Calibration performance was assessed using calibration curves generated through bootstrap resampling (B = 500). These curves compared observed AL incidence with predicted probabilities across risk deciles. Both apparent and bias-corrected calibration curves were generated. The mean absolute error (MAE) between predicted and observed probabilities was calculated as a quantitative measure of calibration. The net clinical benefit across a range of threshold probabilities was evaluated using decision curve analysis (DCA). Model-guided strategies were compared with the “treat-all” and “treat-none” strategies. Standardized net benefit was plotted against threshold probabilities, with corresponding cost-benefit ratios displayed on a secondary axis. Model interpretability was assessed using the SHAP framework at both the global and individual levels. Specifically, (i) global feature importance was quantified using the mean absolute SHAP value (mean |SHAP value|) across all samples; (ii) SHAP summary (beeswarm) plots illustrated the distribution and direction of feature effects, with color gradients representing feature magnitude (high: yellow/orange; low: purple); (iii) SHAP force plots decomposed individual predictions into additive feature contributions, demonstrating how each variable influenced the predicted risk of AL; and (iv) SHAP dependence plots were generated to explore potential nonlinear associations and interaction effects between key predictors and pathological stage.

## 3 Results

### 3.1 Patient basic characteristics

A total of 368 patients with EC who underwent radical esophagectomy were included in the analysis. Patients were randomly assigned to the training cohort (n=258) and validation cohort (n=110). The study population comprised 256 males (70%) and 112 females (30%), with a median age of 63 years (IQR: 58-69). Overall, 102 patients (28%) developed postoperative AL. The incidence of AL was comparable between the training and validation cohorts, with 69 cases (27%) and 33 cases (30%), respectively (**Table 1**).

**Table 1:** Distribution of predictive factors in the training and validation sets.

Variables	Training set (n=258)	Validation set (n=110)	Overall (n=368)	P
Sex				1.000
Male	185 (72)	71 (65)	256 (70)	
Female	73 (28)	39 (35)	112 (30)	
Age	63 (58, 69)	61.5 (57, 67)	63 (58, 69)	0.148
BMI	23.88 (21.8, 25.95)	24.23 (21.97, 27.53)	24.06 (21.86, 26.22)	0.369
Distance	28 (25, 30)	28 (25, 30.75)	28 (25, 30)	0.241
Radio	0.18 (0.16, 0.32)	0.18 (0.16, 0.28)	0.18 (0.16, 0.32)	0.980
Smoking				0.244
No	190 (74)	88 (80)	278 (76)	
Yes	68 (26)	22 (20)	90 (24)	
Drinking				0.587
No	233 (90)	102 (93)	335 (91)	
Yes	25 (10)	8 (7)	33 (9)	
DM				0.205
No	247 (96)	101 (92)	348 (95)	
Yes	11 (4)	9 (8)	20 (5)	
Hypertension				0.504
No	200 (78)	81 (74)	281 (76)	
Yes	58 (22)	29 (26)	87 (24)	
Tstage				0.235
T0-1	54 (21)	17 (15)	71 (19)	
T2	36 (14)	10 (9)	46 (12)	
T3	143 (55)	73 (66)	216 (59)	
T4	25 (10)	10 (9)	35 (10)	
Nstage				0.869
N0	134 (52)	58 (53)	192 (52)	
N1	66 (26)	31 (28)	97 (26)	
N2	45 (17)	17 (15)	62 (17)	
N3	13 (5)	4 (4)	17 (5)	
Mstage				0.557
M0	255 (99)	110 (100)	365 (99)	
M1	3 (1)	0 (0)	3 (1)	
Stage				0.405
I	47 (18)	15 (14)	62 (17)	
II	94 (36)	47 (43)	141 (38)	
III-IV	117 (45)	48 (44)	165 (45)	
Location				0.367
Upper and upper-middle	20 (8)	8 (7)	28 (8)	
Middle	116 (45)	41 (37)	157 (43)	
Lower-middle	83 (32)	37 (34)	120 (33)	
Lower	39 (15)	24 (22)	63 (17)	
Pathological1				0.481
Ulcerative	98 (38)	40 (36)	138 (38)	
Infiltrative	50 (19)	29 (26)	79 (21)	
Protruding	29 (11)	12 (11)	41 (11)	
Others	81 (31)	29 (26)	110 (30)	
Neoadjuvant				0.533
No	202 (78)	90 (82)	292 (79)	
Yes	56 (22)	20 (18)	76 (21)	
Pathological2				0.354
SCC	251 (97)	105 (95)	356 (97)	
ACC	7 (3)	5 (5)	12 (3)	
Grade				0.440
Well-differentiated	22 (9)	14 (13)	36 (10)	
Moderately differentiated	125 (48)	49 (45)	174 (47)	
Poorly differentiated	111 (43)	47 (43)	158 (43)	
HBC	135.5 (124, 146)	133 (120.25, 145)	135 (124, 146)	0.273
RBC	4.47 (4.19, 4.8)	4.49 (4.09, 4.86)	4.48 (4.16, 4.81)	0.931
WBC	5.73 (4.84, 6.86)	5.62 (4.98, 6.71)	5.72 (4.88, 6.78)	0.901
Neutrophils	3.38 (2.62, 4.43)	3.38 (2.9, 4.34)	3.38 (2.68, 4.36)	0.966
Monocytes	0.42 (0.32, 0.52)	0.4 (0.32, 0.5)	0.41 (0.32, 0.51)	0.398
Lymphocytes	1.6 (1.3, 1.98)	1.61 (1.3, 1.92)	1.6 (1.3, 1.96)	0.630
PLT	223.5 (188, 275)	230.5 (197, 282.75)	226 (190.75, 279.25)	0.499
SIRI	0.87 (0.55, 1.32)	0.85 (0.54, 1.23)	0.86 (0.55, 1.31)	0.795
SII	479.51 (318.66, 706.76)	482.56 (352.67, 708.32)	480.13 (319.55, 707.63)	0.692
NLR	2.08 (1.56, 2.87)	2.18 (1.56, 2.97)	2.09 (1.55, 2.89)	0.759
MLR	0.24 (0.19, 0.32)	0.25 (0.19, 0.32)	0.24 (0.19, 0.32)	0.922
PLR	137.31 (107.28, 171.97)	146.53 (118.52, 183.04)	141.34 (111.46, 176.38)	0.243
LNR	0.48 (0.35, 0.64)	0.46 (0.33, 0.64)	0.48 (0.34, 0.64)	0.681
NPAR	0.09 (0.07, 0.11)	0.09 (0.07, 0.11)	0.09 (0.07, 0.11)	0.721
UCr	0.08 (0.06, 0.1)	0.08 (0.06, 0.09)	0.08 (0.06, 0.09)	0.772
Albumin	40.01 (37.87, 42.2)	40.15 (37.65, 41.8)	40.08 (37.85, 42.11)	0.572
ALT	13.5 (10.03, 19.95)	13.2 (9.62, 17.7)	13.4 (9.95, 19.5)	0.420
AST	17.1 (13.83, 20.23)	15.45 (12.93, 21.33)	16.7 (13.5, 20.52)	0.248
ALP	77.7 (65.05, 89.3)	79.5 (69, 89.02)	78.1 (66, 89.3)	0.406
TB	9.8 (7.39, 13.79)	10.3 (7.27, 13.38)	9.8 (7.3, 13.6)	0.744
SCr	65.3 (56.12, 75)	66 (56.9, 71)	65.66 (56.16, 73.4)	0.440
eGFR	95 (88, 101)	95.5 (90, 103.75)	95 (89, 102)	0.335
TC	4.34 (3.82, 5)	4.38 (3.85, 4.89)	4.36 (3.84, 4.96)	0.954
Triglycerides	1.12 (0.86, 1.5)	1.12 (0.97, 1.42)	1.12 (0.9, 1.45)	0.545
Cystatin	0.97 (0.88, 1.08)	0.96 (0.86, 1.09)	0.96 (0.87, 1.08)	0.692
BG	4.82 (4.5, 5.16)	4.8 (4.42, 5.26)	4.82 (4.46, 5.19)	0.987
Na	141.36 ± 2.52	141.84 ± 2.27	141.51 ± 2.45	0.075
K	4.02 ± 0.33	3.94 ± 0.35	4 ± 0.34	0.055
PT	11.9 (11.5, 12.4)	11.9 (11.6, 12.5)	11.9 (11.5, 12.4)	0.161
APTT	27.7 (26.3, 29.1)	27.3 (26.2, 29.08)	27.6 (26.3, 29.1)	0.285
CYFRA21_1	1.54 (1.2, 2.1)	1.53 (1.1, 2.09)	1.54 (1.16, 2.1)	0.681
CEA	2.14 (1.46, 3.08)	2.01 (1.39, 3)	2.09 (1.46, 3.02)	0.536
CA199	4.39 (2, 11.15)	4.57 (2.4, 14.77)	4.46 (2.01, 12.16)	0.237
CA125	11.6 (8.4, 15.85)	12.25 (7.7, 15.2)	11.7 (8, 15.5)	0.893
SCC	0.9 (0.7, 1.3)	1 (0.7, 1.58)	1 (0.7, 1.4)	0.359
Duration	300 (260, 390)	300 (260, 408.75)	300 (260, 400)	0.936
BloodLoss	100 (50, 200)	100 (100, 200)	100 (50, 200)	0.485
Anastomotic leak				0.609
No	189 (73)	77 (70)	266 (72)	
Yes	69 (27)	33 (30)	102 (28)	

(1) Distance: Distance between the tumor and the incisors as measured by gastroscopy; (2) Ratio: Ratio of the tumor-incisor distance (gastroscopy-based) to patient height; (3) TNM stage and tumor location: Classified according to the American Joint Committee on Cancer (AJCC) guidelines.

### 3.2 Independent correlated factors

**Figure 1:**
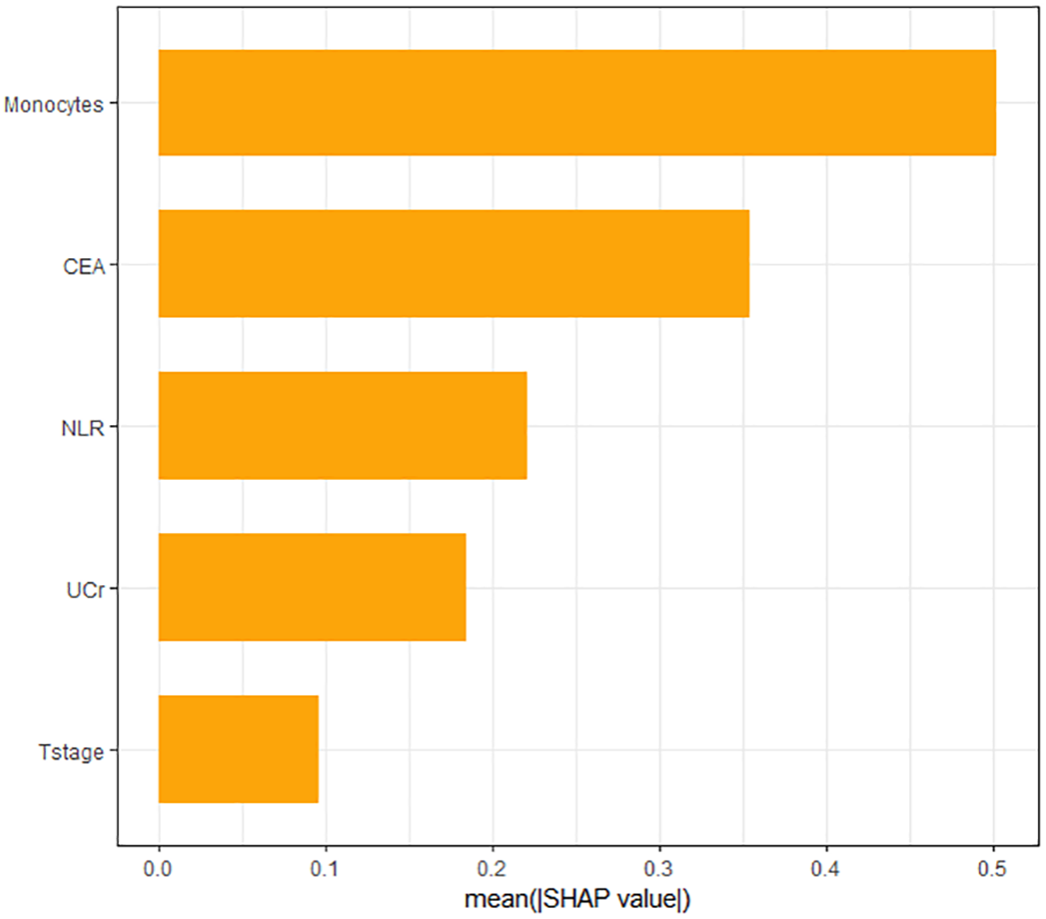
LASSO regression for variable selection.

LASSO regression was performed to identify the optimal predictive variables while minimizing the risk of overfitting. The optimal penalty parameter λ was determined through 10-fold cross-validation. Five variables were ultimately selected for model development: monocyte count, CEA, neutrophil-to-lymphocyte ratio (NLR), UCr, and T stage (**Figure 1**).

### 3.3 Construction of the machine learning model

Five ML models (RF, SVM, LR, XGBoost, LightGBM) were trained on the training set (n=258). Performance was evaluated using AUC, optimal cutoff probability, SEN, and SPE.

**Figure 2:**
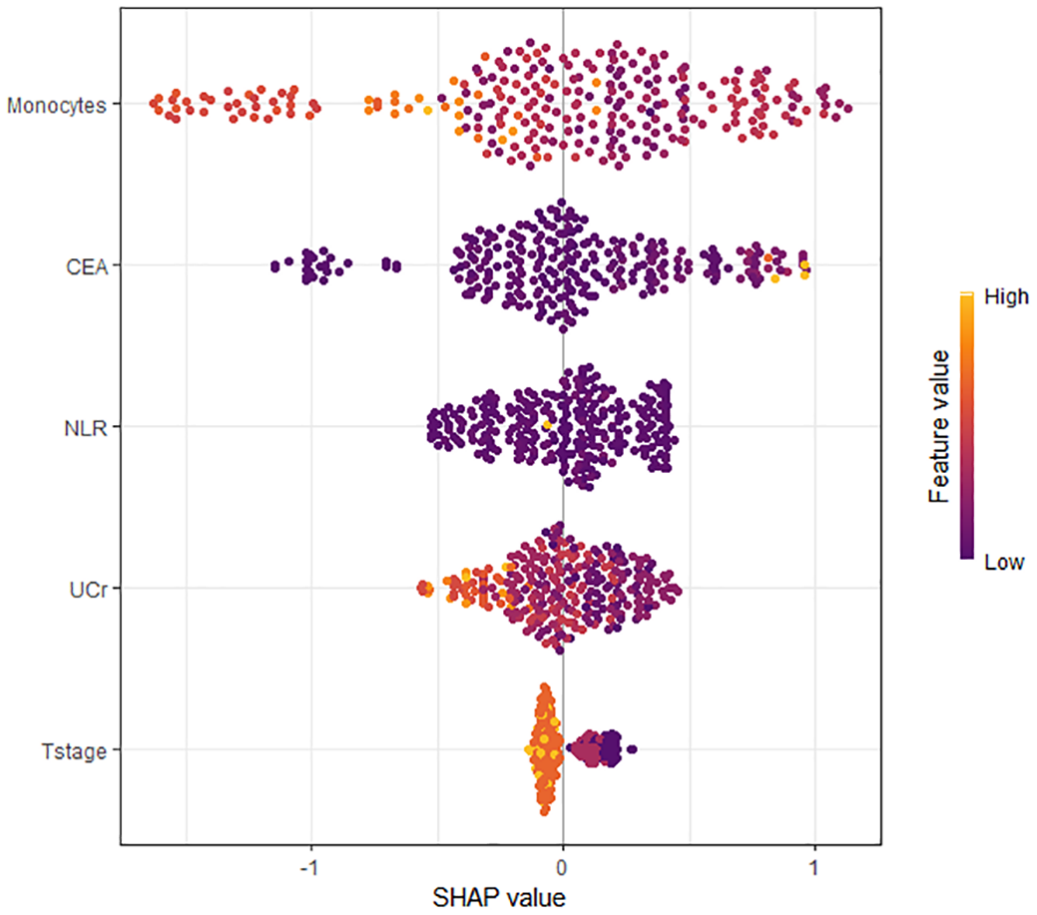
Calibration curve for ML models used to predict AL in the training set.

**Figure 3:**
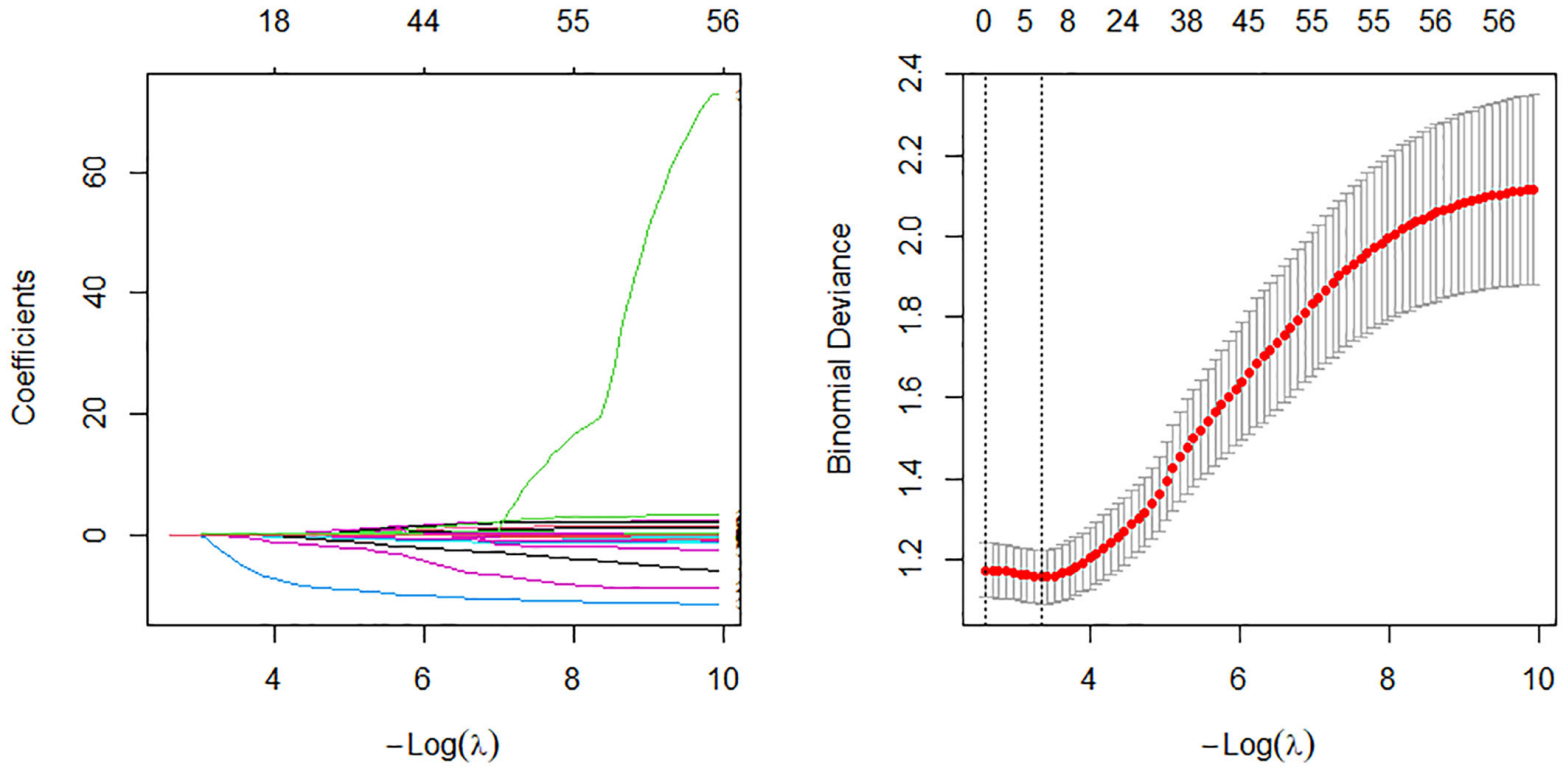
Decision curves for ML models used to predict AL in the training set.

In the training cohort, XGBoost demonstrated the highest discriminative performance (AUC = 0.873, 95% CI: 0.822-0.923), with an optimal cutoff of 0.313, SEN of 0.841, and SPE of 0.804. LightGBM ranked second (AUC = 0.869, 95% CI: 0.820-0.917; cutoff=0.273; SEN = 0.899; SPE = 0.762). RF achieved an AUC of 0.787 (95% CI: 0.711-0.841; SEN = 0.899; SPE = 0.529 at a cutoff of 0.171). SVM yielded an AUC of 0.702 (95% CI: 0.627-0.778; SEN = 0.681; SPE = 0.635). The baseline LR model exhibited the lowest training-set performance (AUC = 0.688, 95% CI: 0.618-0.758; SEN = 0.841; SPE = 0.476) (**Table 2**). Calibration curves (**Figure 2**) demonstrated good agreement between predicted probabilities and observed AL incidence. DCA (**Figure 3**) indicated positive net clinical benefit across a wide range of threshold probabilities.

**Table 2:** Performance of ML models in training and validation sets.

>Model	Training set				Validation set		
	AUC(95%CI)	Threshold	Sensitivity	Specificity	AUC(95%CI)	Sensitivity	Specificity
RF	0.787(0.711-0.841)	0.171	0.899	0.529	0.803(0.717-0.892)	0.879	0.571
SVM	0.702(0.627-0.778)	0.739	0.681	0.635	0.555(0.438-0.670)	0.515	0.468
LR	0.688(0.618-0.758)	0.212	0.841	0.476	0.527(0.401-0.653)	0.606	0.429
XGBoost	0.873(0.822-0.923)	0.313	0.841	0.804	0.723(0.613-0.833)	0.606	0.753
LightGBM	0.869(0.820-0.917)	0.273	0.899	0.762	0.713(0.609-0.816)	0.727	0.623

### 3.4 Model validation

The generalizability of the five models was comprehensively assessed in the independent validation cohort (n=110), with particular emphasis on discriminative performance and clinical applicability. Unlike its performance in the training cohort, RF demonstrated the best discriminative ability and generalizability in the validation cohort, achieving the highest AUC of 0.803 (95% CI: 0.717-0.892). At the optimal cutoff, RF achieved a SEN of 0.879 and a SPE of 0.571.

**Figure 4:**
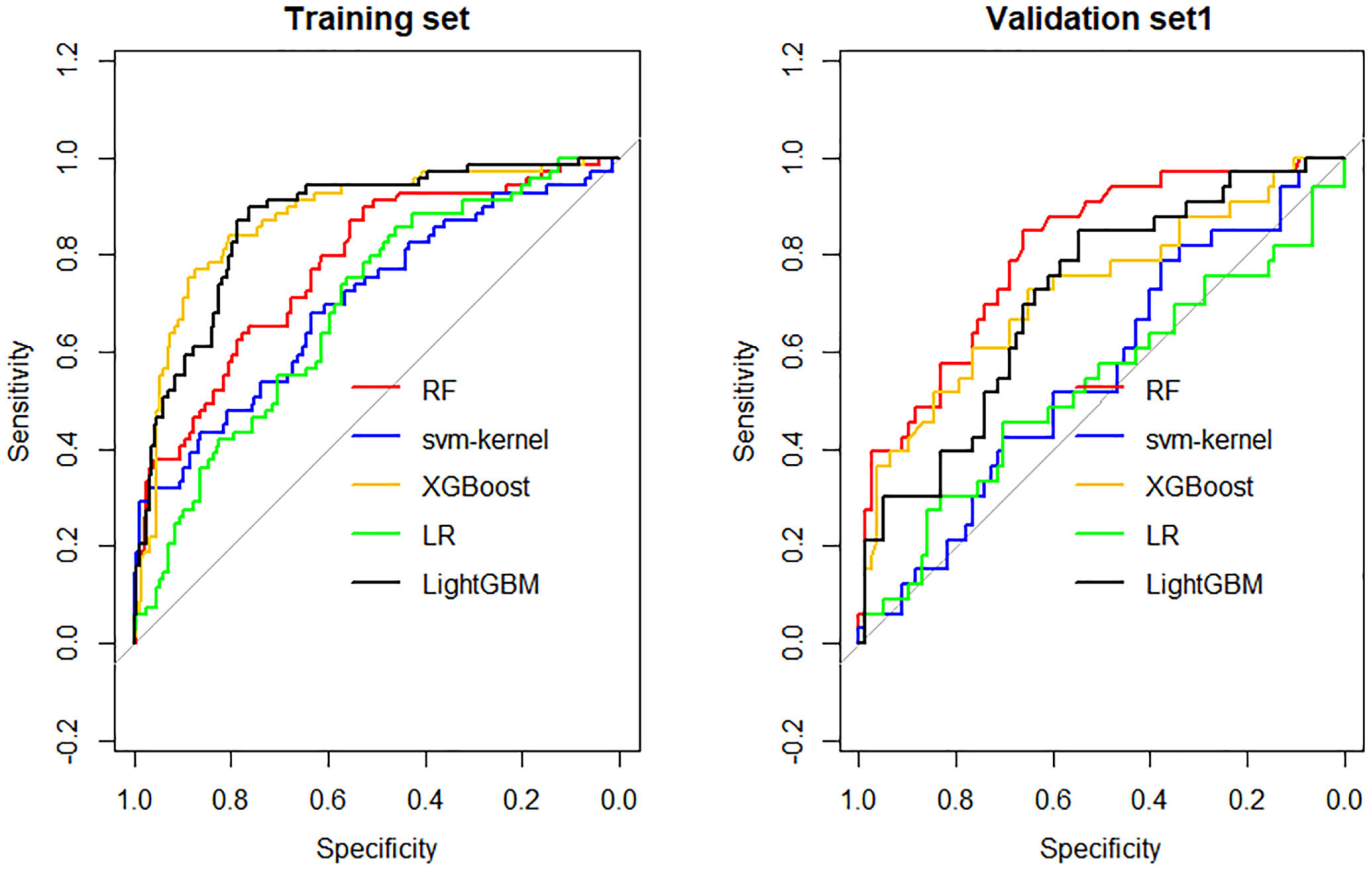
ROC curves of the ML model for predicting AL in training and validation sets.

**Figure 5:**
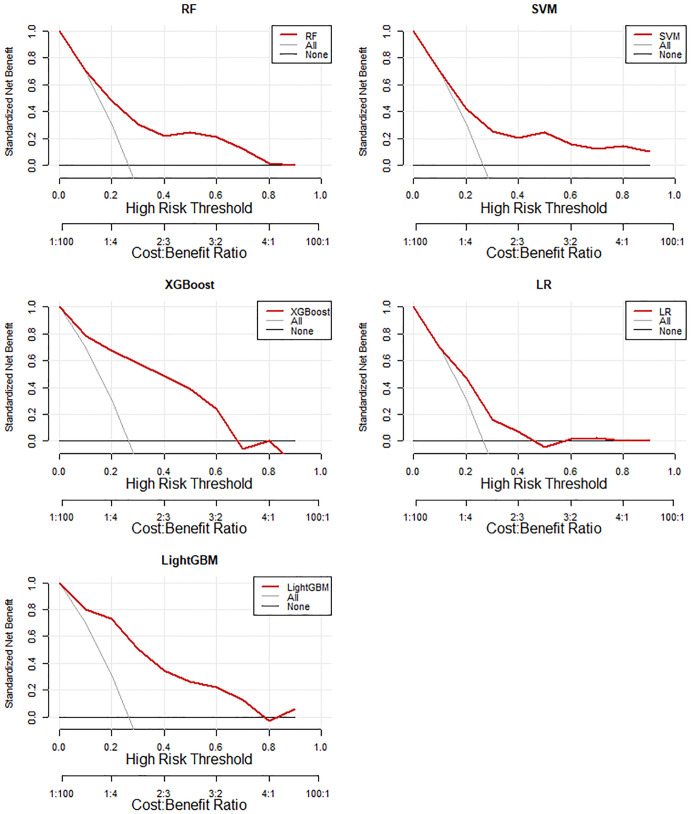
Calibration curve for ML models used to predict AL in the validation set.

**Figure 6:**
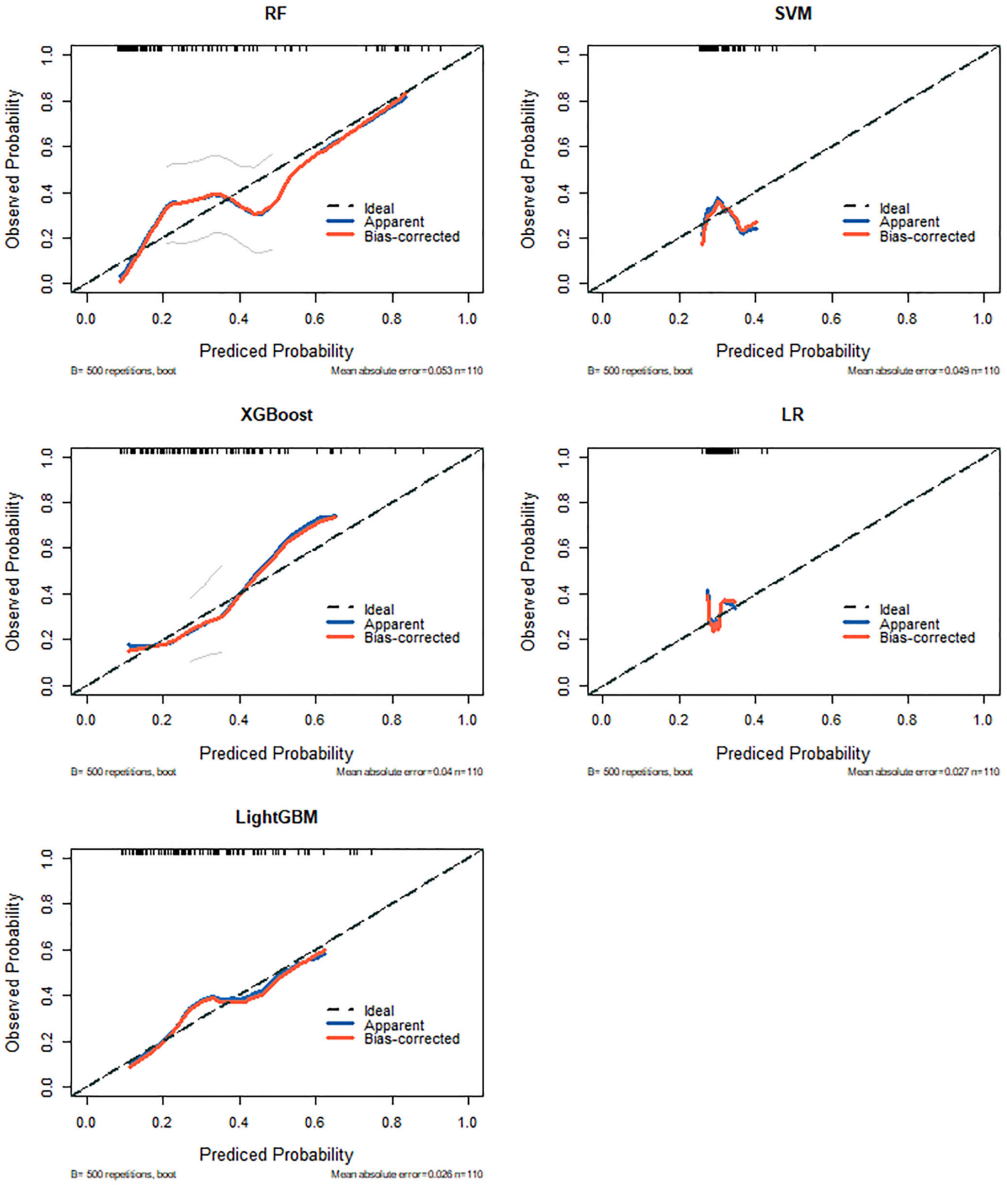
Decision curve for ML models used to predict AL in the validation set.

XGBoost and LightGBM showed moderate predictive performance, with AUCs of 0.723 (95% CI: 0.613-0.833) and 0.713 (95% CI: 0.609-0.816), respectively. In contrast, SVM and LR performed poorly, with AUC values approaching the line of no discrimination (0.555 and 0.527, respectively) (**Table 2**; **Figure 4**). Calibration curves for the validation cohort (**Figure 5**) demonstrated good agreement between predicted and observed outcomes for the ensemble models. DCA (**Figure 6**) further indicated that RF provided greater net clinical benefit for predicting AL than either the “treat-all” or “treat-none” strategy. Accordingly, RF was selected as the optimal predictive model. .

### 3.5 Model interpretability analysis

**Figure 7:**
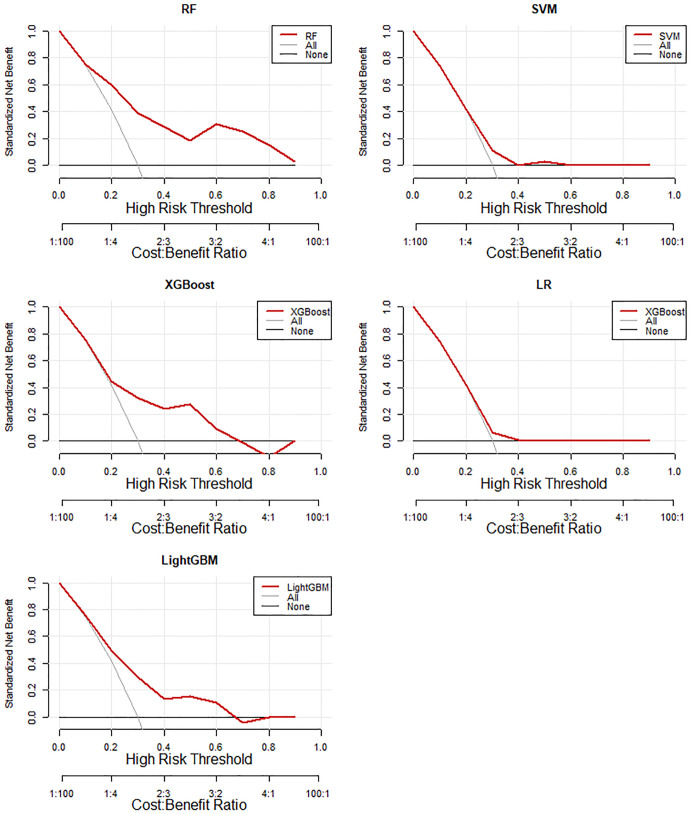
Global feature importance ranking based on SHAP values.

**Figure 8:**
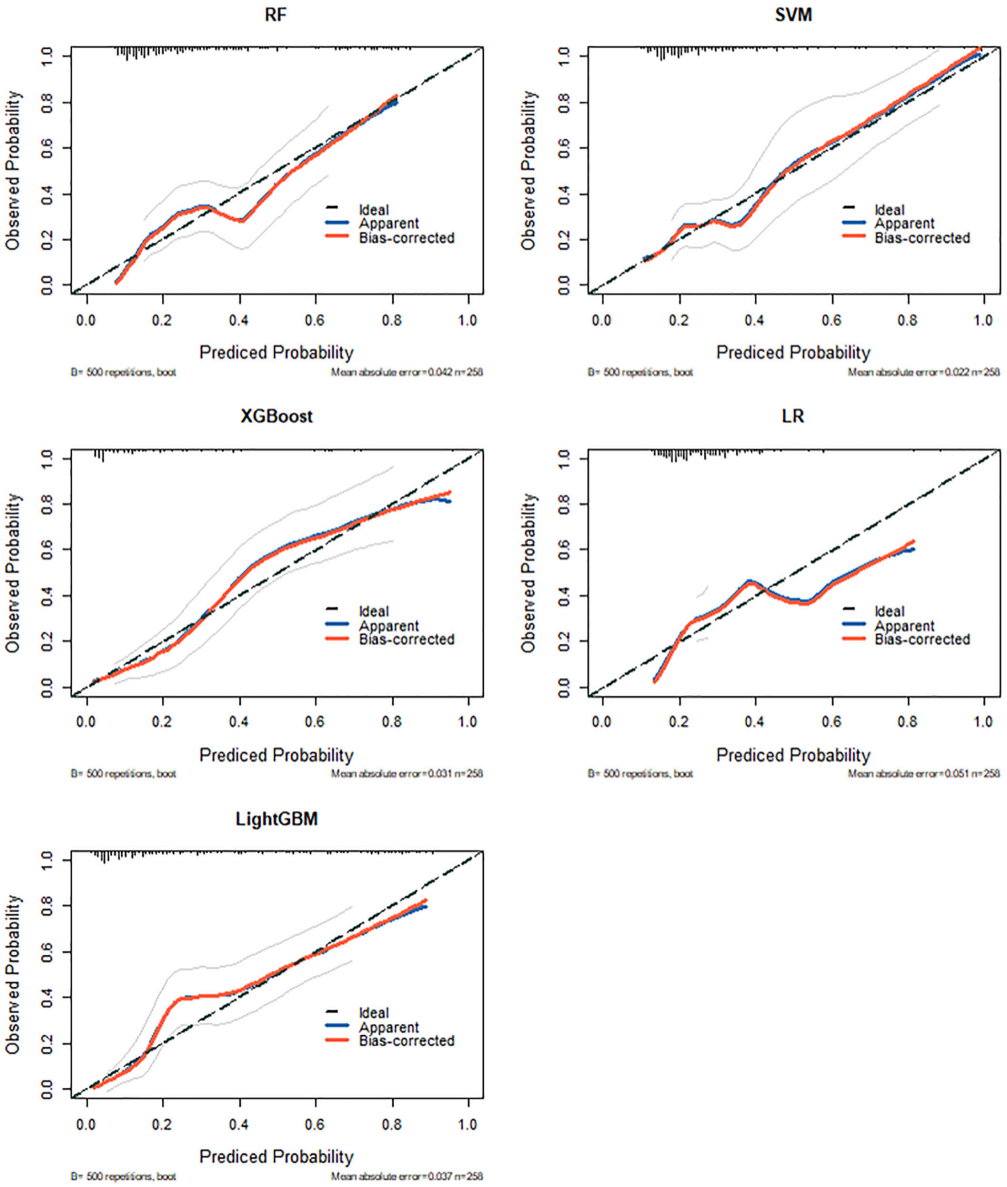
SHAP summary beeswarm plot.

The global feature importance ranking based on SHAP values (**Figure 7**) identified the relative importance of the five predictors. The SHAP summary beeswarm plot (**Figure 8**) illustrated the direction and magnitude of feature effects on model predictions. Higher values of several top-ranked predictors were associated with positive SHAP values, indicating increased AL risk, whereas lower-risk profiles corresponded to negative SHAP values. Furthermore, SHAP dependence plots were generated to investigate potential nonlinear relationships and interaction effects between key predictors and pathological stage. No substantial interaction effects were observed between the principal predictors and pathological stage, supporting the independent predictive contribution of the selected variables.

## 4 Discussion

An ML-based model was developed and validated for predicting in-hospital AL following radical esophagectomy for EC. Among the five algorithms evaluated (RF, SVM, LR, XGBoost, and LightGBM), RF demonstrated the most robust and generalizable predictive performance. In the validation cohort, RF achieved the highest AUC of 0.803 (95% CI: 0.717-0.892), with a SEN of 0.879 and a SPE of 0.571. The high SEN is particularly important in the context of AL prediction, where the consequences of missed cases, including mediastinitis, sepsis, and mortality, substantially outweigh the costs associated with additional postoperative surveillance. In the training cohort, XGBoost and LightGBM achieved the highest AUC values (0.873 and 0.869, respectively). However, both models exhibited substantial declines in performance in the validation cohort (AUCs of 0.723 and 0.713), corresponding to absolute reductions of 0.150 and 0.156, respectively. This pattern is consistent with the well-recognized tendency of boosting algorithms to aggressively minimize training error through iterative residual fitting, thereby capturing noise specific to the training data, particularly when the sample size is moderate relative to model complexity (15). In contrast, RF achieved a training AUC of 0.787 and a validation AUC of 0.803, showing no evidence of performance deterioration. This stability is attributable to the bagging mechanism of RF, whereby multiple decorrelated decision trees are constructed from bootstrap samples, and their predictions are aggregated, thereby reducing variance without substantially increasing bias (16). This mechanism makes RF particularly resilient to overfitting in datasets with moderate sample sizes. Empirical evidence supports that bagging-based ensembles maintain more stable generalization than boosting methods under moderate events-per-variable conditions (17). SVM and LR exhibited poor performance in the validation cohort (AUCs of 0.555 and 0.527, respectively), approaching the line of no discrimination. The limited generalizability of LR is consistent with its inherent assumption of linear relationships between predictors and the log-odds of the outcome, which may oversimplify the multifactorial and nonlinear mechanisms underlying AL development. The poor performance of SVM may reflect its SEN to feature scaling and kernel selection when applied to datasets containing both continuous and categorical variables with a moderate sample size.

An observation that warrants discussion is that the validation AUC of the RF model (0.803) was slightly higher than its training AUC (0.787). Although seemingly counterintuitive, this phenomenon has been reported in the ML literature and may arise from several mechanisms (18). First, the bagging procedure used in RF introduces deliberate randomness through bootstrap sampling and random feature selection at each node split, which may lead to modest underestimation of apparent training performance relative to the model’s true discriminative ability, a phenomenon sometimes referred to as “out-of-bag conservatism.” Second, in a single train-validation split, stochastic variation in the composition of the validation cohort, particularly when the validation sample size is moderate (n=110), may produce AUC estimates that fluctuate around the true population value. The small difference observed (0.016) falls well within the expected range of statistical variation, as evidenced by the substantial overlap between the 95% confidence intervals of the training and validation AUCs. Therefore, this finding does not suggest data leakage or methodological bias but rather reflects the inherent sampling variability associated with single-split validation.

Many ML approaches have been developed to predict AL following esophagectomy. Radiomics-based models have gained increasing attention. Klontzas et al. (19) combined perioperative variables with CT-derived radiomic features using an XGBoost framework and achieved a validation AUC of 0.792, with an SPE of 77.46% and a SEN of 65.22%. Li et al. (20) developed a composite model leveraging enhanced CT radiomic features and clinical data, reporting a validation AUC of 0.856. Yang et al. (21) evaluated nine ML algorithms using clinical and laboratory data and ultimately selected a LightGBM-based model, which achieved a validation AUC of 0.956 after rigorous cross-validation and external validation. Although radiomics-based approaches have demonstrated promising predictive performance, several challenges limit their clinical applicability. First, the impact of heterogeneous imaging acquisition protocols on model reproducibility remains insufficiently investigated. Second, image segmentation is labor-intensive and highly operator-dependent, introducing potential variability. Third, radiomic features are often characterized by high dimensionality and substantial multicollinearity. Despite the use of advanced feature-selection techniques, such as LASSO, minimum redundancy maximum relevance (mRMR), and recursive feature elimination, the risk of overfitting remains a concern when sample sizes are limited. In contrast, the present model relies exclusively on routinely available preoperative clinical and laboratory variables, thereby offering greater interpretability, reproducibility, and ease of implementation across diverse clinical settings.

Through LASSO feature selection and SHAP interpretability analysis, five key predictors of in-hospital AL were identified: monocyte count, CEA, NLR, UCr, and T stage. The events-per-variable ratio in the training cohort was 13.8 (69 events for 5 predictors), exceeding the conventional threshold of 10 recommended for multivariable prediction models (22). This ratio provides sufficient statistical power for stable model development and reduces the risk of overfitting during feature selection. These predictors can be broadly categorized into two pathophysiological domains: systemic inflammatory-immune status and tumor burden. Together, these factors may influence AL risk through their effects on tissue repair capacity, the surgical stress response, and local tissue perfusion.

Monocyte count emerged as the most influential predictor in the SHAP analysis. As key components of the innate immune system, monocytes differentiate into macrophages at the anastomotic site and regulate the inflammatory processes essential for wound healing. However, elevated preoperative monocyte levels may reflect chronic systemic inflammation and immune dysregulation. Excessive infiltration of monocyte-derived macrophages can shift the local microenvironment toward a pro-inflammatory and tissue-destructive state, thereby impairing collagen deposition and angiogenesis at the anastomotic site (23). Furthermore, elevated monocyte counts, a major component of the systemic inflammatory response index (SIRI), have been independently associated with adverse surgical outcomes in patients with gastrointestinal malignancies (24).

NLR, ranked third in importance, is a well-established biomarker reflecting both systemic inflammation and immune competence. Elevated NLR reflects an exaggerated neutrophilic inflammatory response accompanied by relative lymphopenia, indicating impaired adaptive immunity. This imbalance may disrupt the coordinated transition from the inflammatory phase to the proliferative phase of wound healing, which is essential for anastomotic integrity. Neutrophil-derived reactive oxygen species and proteolytic enzymes, including matrix metalloproteinases (MMPs), can directly degrade newly synthesized collagen, whereas lymphocyte depletion may impair the regulatory mechanisms responsible for resolving inflammation (25). Multiple studies have confirmed NLR as an independent predictor of postoperative complications, including AL, in the EC population (26).

CEA, the second most important predictor, does not directly cause AL but may serve as a surrogate marker of tumor burden and biological aggressiveness. Elevated preoperative CEA levels are associated with advanced tumor stage, poor differentiation, and a pro-inflammatory tumor microenvironment. Tumors characterized by high CEA expression frequently exhibit increased secretion of MMPs, which degrade extracellular collagen and may compromise tissue integrity surrounding the anastomosis (27). Additionally, elevated CEA is frequently accompanied by cancer-associated cachexia and chronic inflammation, both of which impair tissue healing capacity (28, 29).

UCr, as an indicator of renal function and metabolic clearance capacity, reflects the body’s ability to eliminate inflammatory mediators and metabolic waste products. Preserved renal function facilitates the clearance of uremic toxins and pro-inflammatory cytokines that may otherwise impair anastomotic healing. Renal dysfunction has been associated with an increased risk of AL through mechanisms including tissue edema, impaired microcirculatory perfusion, and reduced protease inhibitor activity (30, 31).

T stage reflects the depth of tumor invasion and directly influences the extent of surgical resection required. Advanced T stage (T3-T4) generally necessitates more extensive dissection of periesophageal tissues, increasing the risk of gastric conduit devascularization and compromised anastomotic perfusion. Consistent with this mechanism, the SHAP beeswarm plot demonstrated that higher T stage values were associated with positive SHAP values, indicating an increased risk of AL. This finding is in agreement with previous reports identifying local tumor invasion as an important determinant of anastomotic complications (32).

These predictors do not act in isolation but interact synergistically. The RF model, through its ensemble of decision trees, captures these complex, non-linear interactions, for example, the compounding effect of elevated monocytes and high NLR in a patient with advanced T stage, which linear models such as LR cannot represent. This mechanistic synergy partially explains the performance gap between RF and LR observed in our validation set.

### 4.1 Clinical utility and implementation strategy

This study focused on in-hospital AL to ensure diagnostic certainty and maximize data reliability. Based on the optimal probability threshold of 0.171, determined by maximizing Youden’s index in the RF model, a risk-stratified perioperative management strategy was proposed. This threshold should be regarded as a screening threshold rather than a mandatory intervention threshold. For patients with a predicted probability below 0.171, the model effectively excludes in-hospital AL with high SEN (0.879), allowing clinicians to follow standard Enhanced Recovery After Surgery (ERAS) pathways when other clinical findings are reassuring. For patients classified as high risk (predicted probability ≥0.171), intensified postoperative surveillance may be warranted, including maintenance of surgical drains with daily amylase monitoring and performance of contrast-enhanced CT or water-soluble contrast esophagography on postoperative days 5–7 to assess anastomotic integrity before initiation of oral feeding. In resource-limited settings, a higher probability threshold may be adopted to improve specificity and reduce unnecessary imaging investigations. Conversely, institutions implementing accelerated ERAS pathways and early discharge strategies may favor a lower threshold to minimize missed early AL events.

The relatively low threshold of 0.171 reflects the model’s design objective of maximizing SEN in a screening context, where the clinical consequences of missed AL substantially outweigh the costs associated with additional monitoring of false-positive cases. The moderate SPE (0.571) therefore represents an acceptable trade-off, as the downstream interventions applied to high-risk patients, such as prolonged drain retention and additional imaging, are relatively low-risk and inexpensive compared with the morbidity associated with undetected AL.

It should be noted that the present model was developed and calibrated specifically to predict in-hospital AL. In institutions employing accelerated ERAS pathways with median hospital stays of 6–8 days, some subclinical leaks may become clinically apparent only after discharge and therefore fall outside the prediction window of the model. Consequently, prospective validation is required before applying the model in such settings. High-risk patients identified by the model may particularly benefit from extended inpatient observation or structured post-discharge follow-up. The chosen threshold (0.171) prioritizes SEN to minimize missed diagnoses. However, in resource-constrained settings or centers with very short hospital stays, clinicians may opt for a higher threshold (e.g., 0.25-0.30) to improve SPE and reduce unnecessary interventions. A threshold SEN analysis across different clinical contexts would be valuable in future studies.

### 4.2 Limitations of the study

This study has several limitations that should be considered when interpreting the findings. First, this was a single-center retrospective analysis. Although the RF model demonstrated stable performance during internal validation, external validation using independent multicenter cohorts is required to confirm its generalizability across different surgical teams, patient populations, and institutional practices. Second, missing-value imputation was performed before data partitioning. Although the overall proportion of missing data was low and the practical impact was likely limited, this approach may have introduced potential information leakage. Future studies should perform imputation within the training dataset and subsequently apply the imputation model to validation or external cohorts. Third, internal validation relied on a single 70:30 stratified random split rather than repeated cross-validation or bootstrap-based optimism correction. Therefore, performance estimates may be influenced by the specific composition of the validation cohort. In addition, calibration slope and calibration intercept were not reported, limiting the comprehensiveness of calibration assessment. Fourth, post-discharge AL events were not included in the outcome definition. The model is primarily calibrated for early-to-intermediate in-hospital outcomes and may not adequately capture delayed AL events occurring after discharge. Fifth, variability in surgeon experience and technical proficiency was not explicitly modeled and may therefore represent a source of residual confounding. Finally, although the events-per-variable ratio was acceptable, the overall sample size remained moderate. Larger multicenter datasets are needed to improve model robustness and facilitate incorporation of additional clinically relevant predictors, including intraoperative perfusion metrics and nutritional status indicators.

## 5 Conclusion

An RF-based ML model was developed and validated for predicting in-hospital AL following esophagectomy in patients with EC. Using five routinely available preoperative predictors (monocyte count, CEA, NLR, UCr, and T stage), the model demonstrated robust discriminative performance (validation AUC = 0.803) and high SEN (0.879), enabling effective preoperative risk stratification. The proposed risk-stratified management strategy can guide personalized perioperative surveillance and optimize resource allocation. External validation in multi-center cohorts is necessary to confirm generalizability.

## Data Availability

The original contributions presented in the study are included in the article/supplementary material. Further inquiries can be directed to the corresponding author.
